# ID 248 - Eficácia e Segurança do Transplante Multivisceral em Pacientes com Tumores Abdominais, Catástrofes Abdominais e Tromboses Complexas: uma revisão sistemática

**DOI:** 10.5327/2237-9622.2025.v34s1.248

**Published:** 2025-11-25

**Authors:** Bruna Carolina de Araújo, Letícia Aparecida Lopes Bezerra da Silva, Roberta Crevelário de Melo, Cinthia Lanchotte Ferreira, Thaiana Helena Roma Santiago, Antonio Pescuma, Juliana Abud; Alessandra Crescenzi, Alexandre Chagas de Santana, Cláudia Lima Vieira, Inara Pereira da Cunha, Márcia Saldanha Kubrusly, Alex Jones Flores Cassenote, Wallace Breno Barbosa, Luciana Costa Xavier, Luciana Bertocco de Paiva Haddad

**Keywords:** transplante multivisceral, tumores abdominais, catástrofes abdominais, tromboses complexas

## Abstract

**Introdução:**

O transplante multivisceral (TMV) pode ser recomendado como tratamento para alguns casos raros de tumores abdominais, catástrofes abdominais e tromboses complexas do sistema venoso mesentérico portal, sendo o procedimento de TMV, em muitos casos, a única alternativa de restabelecimento da normalidade fisiológica. Dessa forma, este estudo tem como objetivo avaliar a eficácia e a segurança do TMV comparado ao cuidado padrão.

**Método:**

Foi realizada uma revisão sistemática com base nas diretrizes metodológicas nacionais. As buscas por estudos que avaliassem a tecnologia na população de interesse foram realizadas na PubMed, no Embase e na Cochrane Library em 23 de abril de 2024 . O risco de viés foi avaliado por meio das ferramentas da Cochrane e do Instituto Joanna Briggs. O processo de seleção, extração e avaliação da qualidade metodológica dos estudos foi feito em dupla e de modo independente.

**Resultados:**

De 75 publicações identificadas nas bases de dados, após o processo de seleção e elegibilidade de acordo com os critérios de elegibilidade, foram incluídos 12 estudos, sendo três coortes, quatro séries de casos e cinco relatos de caso. Os estudos apresentaram falhas metodológicas quanto à clareza na descrição do método. A sobrevida do paciente foi avaliada em oito estudos, variando de 0% em 3 meses a 100% em 67 meses. A sobrevida do enxerto foi investigada em quatro estudos, variando de 66,7% em 24 meses a 85,7% em 120 meses. A rejeição aguda foi observada em cinco estudos, com variação de 0% em 12 meses a 100% em 6 meses. Um estudo mostrou que 9% dos pacientes realizaram retransplante multivisceral em dois dias. A infecção relacionada à assistência à saúde foi relatada em cinco estudos, variando de nenhuma ocorrência em 12 meses a infecção em todos os pacientes após 54 meses de acompanhamento. Complicações imunológicas e operatórias ocorreram em todos os pacientes entre 1 e 34 meses de acompanhamento. A presença da doença do enxerto contra hospedeiro (DECH) foi relatada em dois estudos, variando de 17% em 9 meses a 33% em 24 meses.

**Conclusão:**

Estudos mostram altas taxas de sobrevida dos pacientes após TMV, com destaque para 100% de sobrevida em dois meses em um dos estudos. No entanto, há registros importantes de perda de enxerto, infecções associadas ao atendimento de saúde, além de complicações imunológicas e cirúrgicas frequentes. A DECH também foi observada em uma proporção significativa dos casos analisados. Deve-se levar em consideração que a maioria dos resultados são provenientes de séries de caso e relatos de caso, em decorrência das condições raras. Por fim, observou-se que o TMV pode aumentar a sobrevida de pacientes com tumores, tromboses difusas e catástrofes abdominais. No entanto, é essencial avaliar cuidadosamente a indicação do paciente para esse tipo de procedimento.

